# The importance of pH adjustment for preventing fibrin glue dissolution in the stomach: an in vitro study

**DOI:** 10.1038/s41598-022-10968-5

**Published:** 2022-04-28

**Authors:** Yoshitaka Takegawa, Toshitatsu Takao, Hiroya Sakaguchi, Tatsuya Nakai, Kazuhiro Takeo, Yoshinori Morita, Takashi Toyonaga, Yuzo Kodama

**Affiliations:** 1 Research & Development Division, Medical Affairs Section, KM Biologics Co., Ltd., Kumamoto, Japan; 2 Division of Gastroenterology, Department of Internal Medicine, Kobe University Graduate School of Medicine, 7-5-2 Kusunoki-cho, Chuo-ku, Kobe, Hyogo Japan; 3 Blood Plasma Product Technical Development Section, Technical Development Department, KM Biologics Co., Ltd., Kumamoto, Japan

**Keywords:** Gastroenterology, Medical research

## Abstract

Combined use of fibrin glue and polyglycolic acid (PGA) sheets has attracted attention as a preventive measure for complications associated with endoscopic submucosal dissection. However, fibrin glue is a protein that may be dissolved by gastric acid. We evaluated the effect of artificial gastric acid on fibrin clot. The dissolution time of three layers of fibrin glue with PGA sheets was measured in five groups (pH 1.2, 2.0, 4.0, 5.5, and 6.0 with pepsin). Measurements of three samples per group were made. The mean number of the remaining layers at each measurement point was observed for 168 h. The time to complete dissolution of the three layers of fibrin gel in the three samples was 2.5 h at pH 1.2, 5 h at pH 2.0, 24 h at pH 4.0, and 48 h and 6 h at pH 5.5. In order to maintain fibrin glue in the stomach for a long period, there was a need to avoid pepsin activation secondary to acidification of gastric juice. The use of strong antacids is recommended.

## Introduction

The use of fibrin glue has been widely investigated in the field of gastrointestinal endoscopy. The treatment method of using fibrin glue and polyglycolic acid (PGA) sheets has recently attracted attention as a preventive method for complications related with gastric endoscopic submucosal dissection (ESD) or gastrointestinal fistula. In fact, some reports suggested the effectiveness of fibrin glue and PGA sheets in fixing ESD-related perforation or gastrointestinal fistula^1–5^ and in preventing delayed bleeding in patients taking antithrombotic drugs^6–8^. The use of fibrin glue as a preventive measure for post-ESD stenosis in the vicinity of the cardia of the stomach is under consideration^9^. Fibrin glue is very compatible with gastrointestinal endoscopy because of its ability to be delivered to the target site as a liquid and its high biocompatibility. Although it is expected to be used in a wide range of applications, the adhesion time of fibrin glue to the target site of the PGA sheet varies among cases. In a report by Fukuda et al., this adhesion time ranged from 1 to 32 days^6^. Because fibrin glue mainly comprises plasma-derived proteins, its use in the stomach carries the risk for dissolution by gastric acid; this may be one of the reasons for the variable effect of fibrin glue. Therefore, in this in vitro study, we investigated the effects of the protein-digesting enzyme pepsin and pH level on fibrin glue.

## Materials and methods

**Figure 1:**
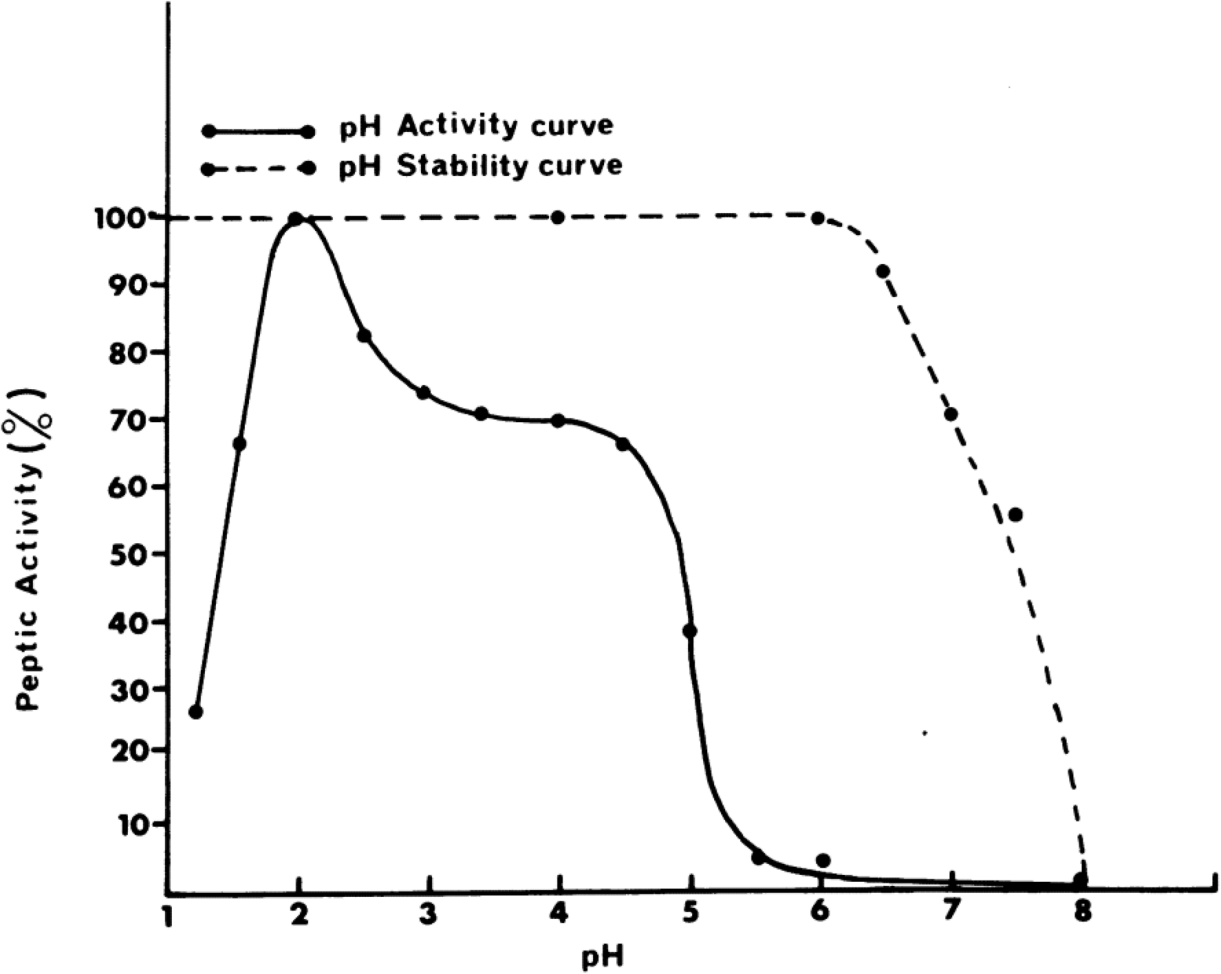
The pH stability and pH activity of pepsin. Quoted from Piper DW, Fenton BH. pH stability and activity curves of pepsin with special reference to their clinical importance.

**Figure 2:**
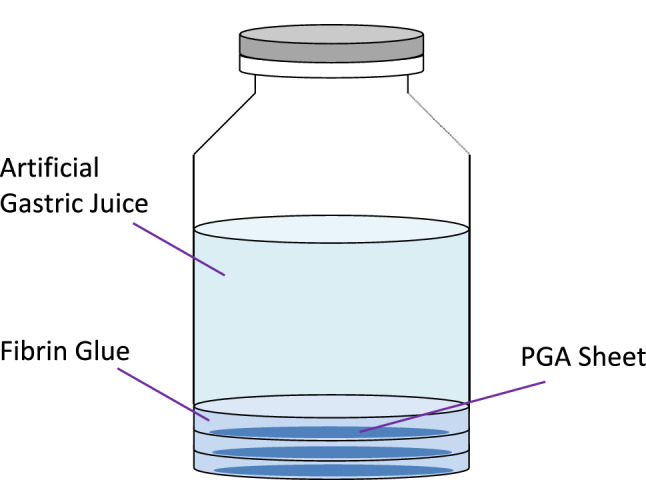
Samples used in this experiment. Three layers of 1-mm fibrin gel and polyglycolic acid sheet are made in the vial (thickness of 3 mm in total). A sample for measurement is prepared by adding 10 mL of the artificial gastric juice.

A PGA sheet measuring 2.3 cm in diameter (NEOVEIL sheet 0.15, GUNZE LIMITED, Kyoto Japan) was placed on the bottom of a glass vial and fixed using fibrin glue (BOLHEAL®, KM Biologics Co., Ltd., Kumamoto Japan). Fibrin glue comprises two solutions (i.e., fibrinogen and thrombin); 0.25 mL of each solution was used to form a 1-mm-thick layer of fibrin glue containing a PGA sheet. This was repeated three times to form a total of three layers per vial. Artificial gastric juice with pepsin was prepared under different conditions of pH1.2, 2.0, 4.0, 5.5, and 6.0; these five groups in total were set up with reference to the activity of pepsin, which peaks at pH 2.0 and drops sharply from pH 4.0 to pH 6.0 (Fig. 1)^11^. As a reference, the dissolution rate of fibrin glue by acid without pepsin was also measured at the same pH levels. Preparation of artificial gastric juice was prepared by adding 1,000 mL of water to 2.0 g of sodium chloride and 7.0 mL of 35% hydrochloric acid, according to the first solution of the JP Dissolution Test Method. In the pepsin-containing group, porcine gastric mucosa-derived pepsin (2,953 U per mg, BBI Solutions Ltd., Cardiff, United Kingdom) was added at 0.1%. The pH was adjusted using sodium hydroxide; hydrochloric acid was also used for fine adjustment. Artificial gastric juice (10 mL) was used for one sample (Fig. 2).

**Figure 3:**
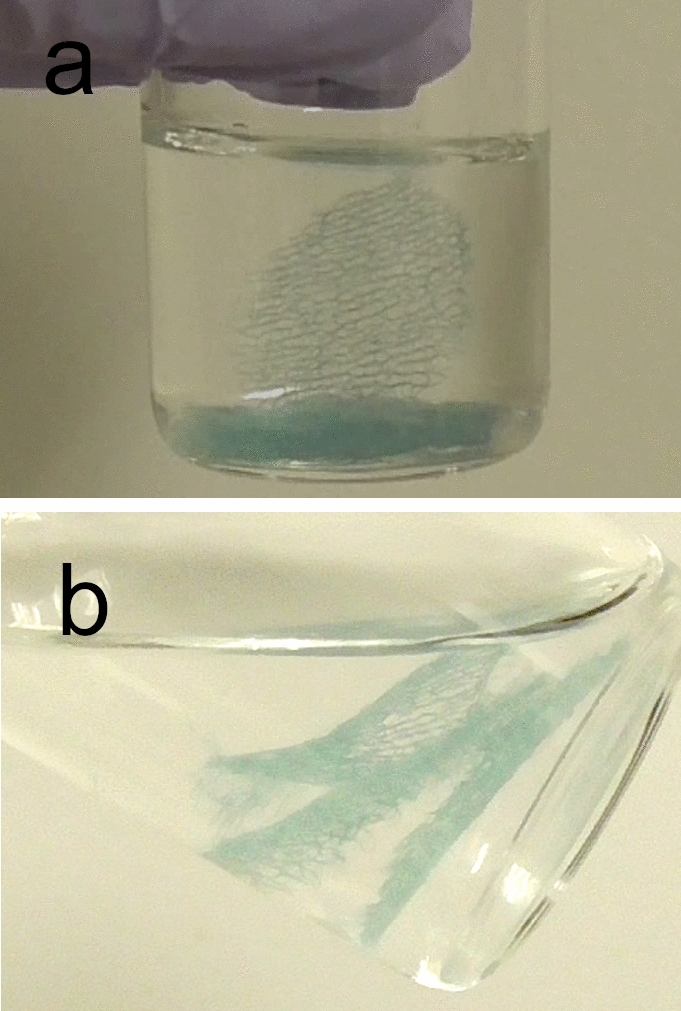
(**a**) The first layer is dissolved. The first PGA sheet is released into the artificial gastric juice during slow shaking. (**b**) All three layers have dissolved, and three PGA sheets are floating in the artificial gastric juice. PGA, polyglycolic acid sheet.

A locking mixer with a shaking condition of 8 rpm (RM-80, AS One Co., Ltd., Osaka, Japan) was installed in an incubator (LST-300D, TOKYO RIKAKIKAI CO, LTD., Tokyo, Japan) and set at 37 °C for the measurements. The mean number of remaining layers was measured every 30 min for the first 6 h, every 6 h until the third day, and every other day until the seventh day; the measurements were compared in three samples per group. As fibrin dissolved, its degradation products and additives contained in the fibrin glue leached into the artificial gastric juice and raised the pH; therefore, the pH was adjusted after each measurement by manual addition of hydrochloric acid. The timing of layer dissolution, which was defined as the release of the PGA sheet into the artificial gastric juice after shaking the vial gently, was recorded (Fig. 3).

## Results

**Figure 4:**
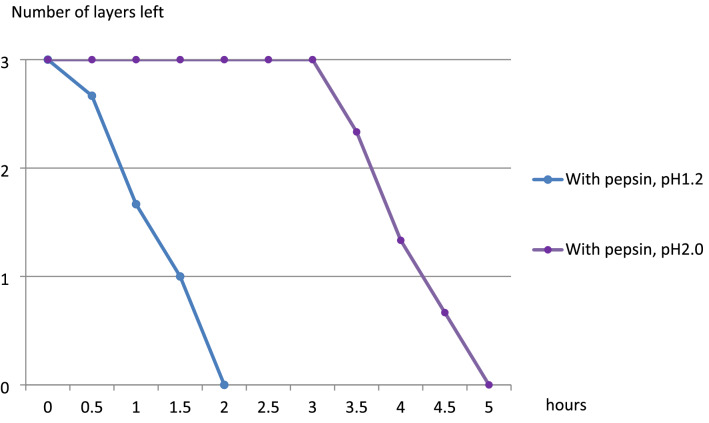
Change in the mean number of the remaining layers in the pH 1.2 and pH 2.0 with pepsin groups over time (N = 3).

**Figure 5:**
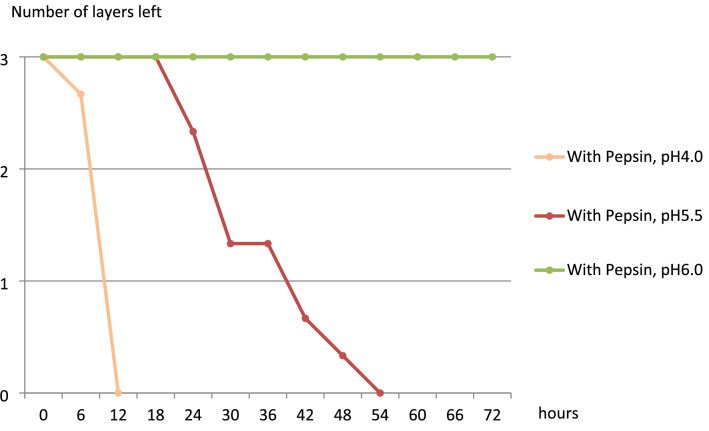
Change in the mean number of the remaining layers in the pH 4.0, pH 5.5, and pH 6.0 with pepsin groups over time (N = 3).

**Figure 6:**
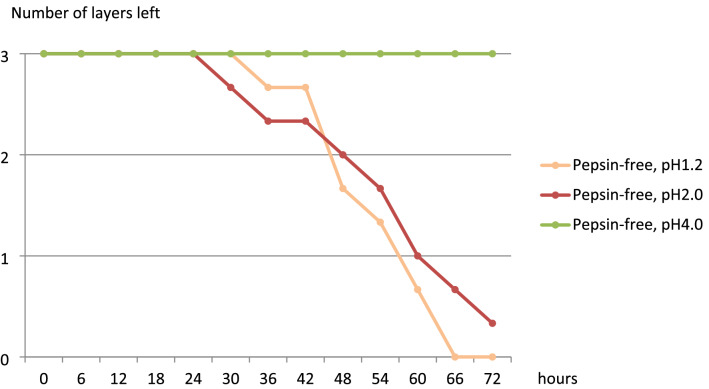
Change in the mean number of the remaining layers in the pH 1.2, pH 2.0, and pH 4.0 pepsin-free groups over time (N = 3).

All three layers of fibrin in all three samples with pepsin dissolved in 2 h in the pH 1.2 group, in 5 h in the pH 2.0 group, in 12 h in the pH 4.0 group, and in 48 h and 6 h in the pH 5.5 group (Figs. 4 and 5). In the pH 6.0 with pepsin group, fibrin was not dissolved at all, even after 168 h. In the pepsin-free groups, all three layers of samples disappeared completely after 72 h in the pH 1.2 group and after 96 h in the pH 2.0 group; in the pH 4.0 group (Fig. 6), fibrin was not dissolved at all even after 168 h.

## Discussion

The present study showed that fibrin glue was dissolved by gastric acid, but its dissolution could be significantly prevented by controlling the pH level. Our results suggested that fibrin glue can be easily dissolved by gastric juice under low pH conditions. Although the activity of pepsin is the highest at pH 2.0 and is reduced to less than 30% at pH 1.2^10^, the relatively fast dissolution of fibrin in the pH 1.2 group was likely brought about by cleavage of the fibrin gel fibers by the strong acid itself. Dissolution of fibrin glue was more delayed in pH 4.0 than in pH 1.2 and 2.0, requiring 24 h for complete dissolution. Although 70% of pepsin activity remains at pH 4.0^10^, it was likely that the delay in dissolution was secondary to inhibition of protein cleavage by the acid. When the pH was raised to 5.5, pepsin activity dropped to below 10%^10^, and fibrin glue dissolution in all samples took 54 h. Moreover, at pH 6, the fibrin glue did not dissolve for 168 hoursin any of the layers in all samples. These results implied the importance of keep the pH high to prevent fibrin dissolution in order to both counteract the effect of the acid and inhibit the activity of pepsin.

Vonoprazan is a potassium antagonist that has been shown to maintain an acid-blocking effect that is higher than that of the proton pump inhibitors esomeprazole and rabeprazole in healthy adult men; after a daily dose of 20 mg for seven days, it was found to have a retention rate of > 85% at pH 4 or higher for 24 h^11^. The other advantage of vonoprazan is that it provides maximal acid-inhibition from the first day of administration^11^, whereas PPIs require several doses and have a slower effect on gastric acid secretion, reaching a plateau in 3–5 days^12,13^. Some comparative studies on the treatment of artificial ulcers after ESD have shown that vonoprazan had a stronger acid-inhibiting effect than PPIs^14,15^. The use of vonoprazan may be effective in prolonging the adhesive effect of fibrin glue in the stomach.

However, the extent to which the use of vonoprazan affects delayed bleeding rates in practice is unknown. Tsuji et al. reported a successful reduction in the rate of delayed bleeding with this treatment despite the use of rabeprazole^16^. In our opinion, many factors determine the effectiveness of this treatment method, and control of intragastric pH is only one of them. Therefore, it is necessary to accumulate various innovations.

Although the combination of fibrin glue and PGA sheets has been used for a long time in the surgical field, its basic research and use in clinical practice in the field of gastrointestinal endoscopy had been few. When this strategy used during gastrointestinal endoscopy, it would be necessary to consider issues, such as limited manipulation through the forceps hole and the abundant amount of mucus and digestive juices in the operative field, compared with that in the surgical field. In previous basic experiments, PGA sheets exposed to highly viscous liquids, such as saliva and gastric mucus, was proven to prevent the adhesion of fibrin glue^17,18^. To solve this problem, we developed and showed the usefulness of a delivery system for PGA sheets in the gastrointestinal tract through animal experiments and excised pig stomach^18–20^. Based on this present study, we propose the use of a strong antacid agent to extend the effectiveness of fibrin glue in the gastrointestinal tract. Although accumulation of clinical experience is important, we believe that this additional basic study would strengthen the value of this technique in dealing with complications of gastrointestinal endoscopic procedures.

## Limitations

The values shown in the results of this study are the averages of three samples from each group conducted in the exploratory research. The in vitro design of this study was less ideal for observing fibrin dissolution, compared with that of the actual environment in the stomach for two reasons. First, the constant immersion in artificial gastric acid and continuous shaking of fibrin gel resulted in uninterrupted exposure to acid and pepsin. Second, the absence of viscous components, such as mucin, in the artificial gastric acid made the fibrin gel highly permeable. Fukuda et al. reported that PGA sheets fixed with fibrin glue remained in the stomach for up to 32 days^6^; therefore, the results of the present study cannot be applied to clinical practice in the time period. In addition, in clinical practice, the fibrin glue should remain in the stomach longer because stomach acid is diluted by eating and drinking water. Care must be taken to not overestimate gastric acid dissolution of the fibrin glue. The actual impact on patients needs to be verified in clinical trials.

## Conclusions

Keeping a near neutral pH in the stomach was important to prevent gastric acid dissolution of fibrin glue. Selection of a strong antacid may be effective in prolonging the duration of fibrin glue retention in the stomach.

## Supplementary Information

Supplementary Information.

## Data Availability

The datasets used and/or analyzed during the current study are available from the corresponding author on reasonable request.
