# Supplementary material for: The importance of pH adjustment for preventing fibrin glue dissolution in the stomach: an in vitro study
Source: Sci Rep. 2022 Apr 28;12:6986. doi: 10.1038/s41598-022-10968-5 (PMC9050883; doi:10.1038/s41598-022-10968-5)
Supplement: Supplementary file 1 — Supplementary Information. [file 41598_2022_10968_MOESM1_ESM.pdf]

Number of layers remaining

	With Pepsin			Pepsin-free			With Pepsin			Pepsin-free			With Pepsin			Pepsin-free			With Pepsin			Pepsin-free			With Pepsin			Pepsin-free			With Pepsin			Pepsin-free		
pH	1.2			1.2			2			2			4			4			5.5			5.5			6			6			7			7		
30min	3	3	3	3	3	3	3	3	3	3	3	3	3	3	3	3	3	3	3	3	3	3	3	3	3	3	3	3	3	3	3	3	3	3	3	3
60min	2	3	3	3	3	3	3	3	3	3	3	3	3	3	3	3	3	3	3	3	3	3	3	3	3	3	3	3	3	3	3	3	3	3	3	3
90min	1	2	2	3	3	3	3	3	3	3	3	3	3	3	3	3	3	3	3	3	3	3	3	3	3	3	3	3	3	3	3	3	3	3	3	3
120min	1	1	1	3	3	3	3	3	3	3	3	3	3	3	3	3	3	3	3	3	3	3	3	3	3	3	3	3	3	3	3	3	3	3	3	3
150min	0	0	0	3	3	3	3	3	3	3	3	3	3	3	3	3	3	3	3	3	3	3	3	3	3	3	3	3	3	3	3	3	3	3	3	3
180min				3	3	3	3	3	3	3	3	3	3	3	3	3	3	3	3	3	3	3	3	3	3	3	3	3	3	3	3	3	3	3	3	3
210min				3	3	3	2	2	3	3	3	3	3	3	3	3	3	3	3	3	3	3	3	3	3	3	3	3	3	3	3	3	3	3	3	3
240min				3	3	3	1	1	2	3	3	3	3	3	3	3	3	3	3	3	3	3	3	3	3	3	3	3	3	3	3	3	3	3	3	3
270min				3	3	3	1	0	1	3	3	3	3	3	3	3	3	3	3	3	3	3	3	3	3	3	3	3	3	3	3	3	3	3	3	3
300min				3	3	3	0		0	3	3	3	3	3	3	3	3	3	3	3	3	3	3	3	3	3	3	3	3	3	3	3	3	3	3	3
330min				3	3	3				3	3	3	3	3	3	3	3	3	3	3	3	3	3	3	3	3	3	3	3	3	3	3	3	3	3	3
360min				3	3	3				3	3	3	3	3	2	3	3	3	3	3	3	3	3	3	3	3	3	3	3	3	3	3	3	3	3	3
12h				3	3	3				3	3	3	2	2	2	3	3	3	3	3	3	3	3	3	3	3	3	3	3	3	3	3	3	3	3	3
18h				3	3	3				3	3	3	1	1	1	3	3	3	3	3	3	3	3	3	3	3	3	3	3	3	3	3	3	3	3	3
1D				3	3	3				3	3	3	0	0	0	3	3	3	2	2	3	3	3	3	3	3	3	3	3	3	3	3	3	3	3	3
1D6h				3	3	3				3	2	3				3	3	3	1	1	2	3	3	3	3	3	3	3	3	3	3	3	3	3	3	3
1D12h				3	3	2				2	2	3				3	3	3	1	1	2	3	3	3	3	3	3	3	3	3	3	3	3	3	3	3
1D18h				2	2	1				2	2	3				3	3	3	0	1	1	3	3	3	3	3	3	3	3	3	3	3	3	3	3	3
2D				2	2	1				2	1	3				3	3	3	0	0	1	3	3	3	3	3	3	3	3	3	3	3	3	3	3	3
2D6h				2	2	0				1	1	3				3	3	3			0	3	3	3	3	3	3	3	3	3	3	3	3	3	3	3
2D12h				1	1					1	0	2				3	3	3				3	3	3	3	3	3	3	3	3	3	3	3	3	3	3
2D18h				0	0					0		2				3	3	3				3	3	3	3	3	3	3	3	3	3	3	3	3	3	3
3D												1				3	3	3				3	3	3	3	3	3	3	3	3	3	3	3	3	3	3
4D												0				3	3	3				3	3	3	3	3	3	3	3	3	3	3	3	3	3	3
5D																3	3	3				3	3	3	3	3	3	3	3	3	3	3	3	3	3	3
6D																3	3	3				3	3	3	3	3	3	3	3	3	3	3	3	3	3	3
7D																3	3	3				3	3	3	3	3	3	3	3	3	3	3	3	3	3	3
